# Comment on “A positive spin: large language models can help directors evaluate programs through their patients' own words”

**DOI:** 10.1097/PR9.0000000000001351

**Published:** 2025-12-19

**Authors:** Hinpetch Daungsupawong, Viroj Wiwanitkit

**Affiliations:** a Private Academic Consultant, Phonhong, Lao People's Democratic Republic; b Department of Research Analytics, Saveetha Dental College and Hospitals, Saveetha Institute of Medical and Technical Sciences Saveetha University, Chennai, India

We would like to comment on the publication on “A positive spin: large language models can help directors evaluate programs through their patients' own words.”^1^ This study looked into the ability of large language models (LLMs) like ChatGPT to interpret qualitative remarks from participants in a pain education program. The study used both LLM-based and manual theme review methodologies to illustrate the viability of using LLMs to assess patient responses. Although the use of LLMs for qualitative research is a promising advance, some areas need to be explored further.

First, although the thematic analysis found 7 major motifs, it does not detail how the LLMs were trained or calibrated to accomplish this task efficiently. Large language models are inevitably influenced by the data used for training, and ChatGPT may struggle with context-specific language or delicate patient interactions. For example, patient reactions during pain education may have complicated emotional connotations and cultural connections that LLMs might not completely get. How did the authors guarantee that the LLMs were calibrated correctly for this domain, and what procedures did they take to minimize potential bias and misunderstanding?

Second, despite the study's reference to a “dual analysis methodology,” there is no information about the procedure and criteria for comparing the outcomes of the LLM-based and manual analyses. How did theme reviewers manually go through the results generated by ChatGPT? Were there any major differences between the 2 methods? If so, how did they get adjusted? Interrater reliability between LLM and human reviewers is a crucial consideration that should be thoroughly explained to demonstrate the robustness of the findings.

Regarding innovation, this study presents a fresh technique to accelerating qualitative analysis with LLM, which is especially relevant in large-scale investigations and resource-constrained contexts. However, future research might look into the unique benefits and drawbacks of LLM against classic qualitative approaches such in-depth interviews and self-coding. One promising option is to improve LLM to detect more subtle patterns, such as emotional tone and patient motivation, to boost the depth of qualitative insights.

In the future, incorporating LLM into clinical and research contexts may enable more scalable and efficient processing of patient feedback, allowing for real-time modifications to therapy and program delivery. However, future study should take into account ethical concerns, such as data privacy and potential algorithmic prejudice. Further study will look into ways to train LLM on a variety of patient-specific datasets to boost sensitivity to individual characteristics and make it a more dependable and empathetic tool in health care.

## Disclosures

The authors have no conflict of interest to declare.
